# Nutritional and Physiological Properties of *Thymbra spicata*: In Vitro Study Using Fecal Fermentation and Intestinal Integrity Models

**DOI:** 10.3390/nu16050588

**Published:** 2024-02-21

**Authors:** Mohamad Khalil, Francesca Piccapane, Mirco Vacca, Giuseppe Celano, Laura Mahdi, Valeria Perniola, Carmen Aurora Apa, Alessandro Annunziato, Ilaria Iacobellis, Giuseppe Procino, Maria Calasso, Maria De Angelis, Rosa Caroppo, Piero Portincasa

**Affiliations:** 1 Clinica Medica “A. Murri”, Department of Precision and Regenerative Medicine and Ionian Area (DiMePre-J), University of Bari Medical School, 70124 Bari, Italy; mohamad.khalil@uniba.it (M.K.); lauramahdi5@gmail.com (L.M.); valeriaperniola17@gmail.com (V.P.); 2 Department of Bioscience, Biotechnology and Environment, University of Bari, 70125 Bari, Italy; francesca.piccapane@uniba.it (F.P.); giuseppe.procino@uniba.it (G.P.); rosa.caroppo@uniba.it (R.C.); 3 Department of Soil, Plant and Food Sciences, University of Bari Aldo Moro, Via Amendola 165/a, 70126 Bari, Italy; mirco.vacca@uniba.it (M.V.); giuseppe.celano@uniba.it (G.C.); carmen.apa@uniba.it (C.A.A.); alessandro.annunziato@uniba.it (A.A.); ilaria.iacobellis@uniba.it (I.I.); maria.calasso@uniba.it (M.C.); maria.deangelis@uniba.it (M.D.A.)

**Keywords:** *Thymbra spicata*, carvacrol, gut microbiota, intestinal permeability, polyphenols

## Abstract

(Poly)phenolic-rich Mediterranean plants such as *Thymbra spicata* have been associated with several health-promoting effects. The nutritional value, as well as physiological interaction of *T. spicata* with the gastrointestinal tract, has not been investigated before. The nutritional composition of *T. spicata* leaves was here characterized by standard analytical methods. *T. spicata* leaves were subjected to ethanolic extraction, simulated gastrointestinal digestion, and anaerobic microbial gut fermentation. Phenols/flavonoid contents and radical scavenging activity were assessed by colorimetric methods. The volatile organic compounds (VOCs) were detected by gas chromatography coupled with mass spectrometry. The effect on intestinal integrity was evaluated using a Caco-2 monolayers mounted in a Ussing chamber. *T. spicata* contains a high amount of fiber (12.3%) and unsaturated fatty acids (76% of total fat). A positive change in VOCs including short-chain fatty acids was observed without significant change in viable microbe. *T. spicata* and carvacrol (main phenolic compound) enhanced ionic currents in a concentration-dependent manner without compromising the Caco-2 monolayer’s integrity. These effects were partially lost upon simulated digestion and completely abolished after colonic fermentation in line with polyphenols and carvacrol content. Conclusion: *T. spicata* represents a promising nutrient for the modulation of gut microbiota and the gut barrier. Further studies must better define its mechanisms of action.

## 1. Introduction

Dietary components and eating patterns can affect important health outcomes. Evidence points to a close link between dietary pattern and decreased mortality [1]. Specific factors that can influence the choices include personal preference, gender, social and cultural aspects, as well as economic considerations. As underscored by several meta-analyses and societies, including the WHO [2], the American Institute for Cancer Research [3], and the US Department of Agriculture [4], a high-quality and healthy diet rely on the limited consumption of saturated and industrial trans fats, red and processed meats, sugar, sodium, and alcohol. By contrast, the consumption of fruits, vegetables, legumes, nuts, and whole grains is greatly encouraged. The Mediterranean diet (MD) mirrors several of the above-mentioned beneficial features, since it is based on the consumption of vegetables, fruits, nuts, olive oil, and many herbs. Besides the richness of MD in essential nutrients, vegetables and herbs contain a high amount of polyphenols [5]. Polyphenols are a diverse group of naturally occurring compounds linked to various health-promoting effects. The gut–polyphenols interaction occurs after the ingestion of food, where polyphenols are modified by digestive enzymes [6]. Small amounts of polyphenols are absorbed in the small intestine, while the greatest amount remains unabsorbed and accumulates in the colon, where it is extensively metabolized by gut microbiota prior to reaching the circulation [7,8,9]. Information about polyphenol biotransformation, bioavailability, and in vivo physiological effects is still yet to be delineated. 

Since the gut microbiota play a pivotal role in host homeostasis and energy balance, changes in their composition can be associated with disease states through the promotion of immune-mediated inflammatory disorders and increasing the intestinal permeability, ultimately leading to the impairment of intestinal barrier function. Evidence from pre-clinical and clinical studies suggests that polyphenols or polyphenol-rich food can positively influence gut health by promoting the growth of beneficial gut bacteria and suppressing the growth of harmful bacteria. This can contribute to a balanced gut microbiome and gut barrier [7]. 

Za’atar is one of the most popular plant-based foods in the Eastern Mediterranean region. Za’atar is a mixture of different thyme-like plant leaves including *Origanum syriacum* and *Thymbra Spicata*, fruits of *Rhus coriaria* (Sumac), and sesame seeds [10]. The mixture contains hundreds of antioxidant compounds, especially polyphenols. Za’atar compounds have been studied from a gastrointestinal perspective, concerning both gut microbiota and gastrointestinal diseases, suggesting that Za’atar polyphenols may provide beneficial effects in the complex interplay between the diet, GM, and intestinal permeability [11].

In Lebanon, *Thymbra spicata* is widely diffused. It belongs to the *Lamiaceae* family and it is rich in polyphenols such as rosmarinic acid, carvacrol (CVL) and different flavonoids [10]. *T. spicata* leaves and flowers are used as food ingredients in salad and in tea infusions. Different biological and bio-functional effects have been reported for *T. spicata* including anti-oxidant and antisteatotic [12], antihypercholesterolaemic [13,14], antitumoral [15], antiviral [16], and antimicrobial [17,18,19] activities. 

CVL is a monoterpenoid phenolic compound found in the essential oils of various plants, particularly in the *Lamiaceae* family. *T. spicata* contains up to 80% CVL in its essential oil [20]. CVL has gained attention among the scientific community due to its diverse biological activities and its potential health benefits [21,22,23]. CVL exerts antimicrobial [24], antioxidant [25], anti-inflammatory [26], and antisteatotic effects [27], and is being explored for various pharmacological and nutraceutical applications.

To our knowledge, no studies have reported the effects of *T. spicata* on the gastrointestinal tract. Based on pre-clinical studies concerning compounds found in *T. spicata*, we have theorized that *T. spicata* may have potential nutraceutical applicability for disorders involving the gastrointestinal tract and gut–liver axis. The translational value of such studies would be of major interest. The objective of this research is to investigate the nutritional profile, physiological implications, and bioactive properties of *T. spicata*, particularly emphasizing the interest in simulating in vitro gastrointestinal digestion and microbial fermentation. This approach allows for a comprehensive exploration of how *T. spicata* affects gut microbiota composition and metabolites. Additionally, the study aims to evaluate the impact of *T. spicata* on the intestinal epithelial function, including permeability and electrophysiological characteristics.

## 2. Methods

### 2.1. Plant Collection and Extraction

Leaves of *Thymbra spicata* L. were collected from flowering plants growing in “Maarakeh”, South Lebanon, during June 2022. The voucher specimen (L1.125/1) was authenticated by Prof. G. Tohme (CNRS, Beirut, Lebanon). The solvent extraction method was used for ethanolic extraction, as described previously by Khalil et al. [15].

### 2.2. Nutritional Value of Thymbra spicata 

The nutritional value of *T. spicata* leaves was determined by Food Safety LAB s.r.l. (Corato, BA, Italy) accredited and certified by international standards and requirements.

The energy value (Kcal and kJ) was determined according to Regulation (EU) No. 1169/2011. Sodium chloride was measured according to the L-MI068 method. Carbohydrates were calculated as total sugar content (DLgs n° 77 16/02/1993 GU n° 69 24/03/1993), dietary fiber (according to AOAC 985.29), and simple sugars (L-MI014 rev.0 2016). Total fat was calculated according to DM 23-07-1994 SO n°4 G.U. n°186 del 10/08/1994 and single fatty acids were calculated by the AOCS Ce 2-66:2009 + AOCS Ce 1e-91:2001 method. The total proteins content was calculated by UNI CEN ISO/TS 16634-2:2016. Ashes was evaluated via ISO 2171:2007, and humidity was calculated using UNI EN ISO 712:2010.

### 2.3. Simulated Digestion and Colonic Fermentation

#### 2.3.1. In Vitro Simulation of Gastrointestinal Digestion

Simulated *T. spicata* digestion was carried out in vitro using different solutions mimicking the oral, gastric, and intestinal fluids [28]. Briefly, 5 g of dried *T. spicata* leaves and distilled water (25 mL) were homogenized for 3 min with a lab stomacher (Bag Mixer, Interscience International; Roubaix, France). The oral phase accounted for the addition of 10 mg of α-amylase dissolved in 3.125 mL of 1 mM CaCl_2_ solution, and samples were incubated for 30 min at 37 °C under stirring conditions (50 rpm). To simulate gastric fluid, 1.35 mg of pepsin was dissolved in 12.5 mL of 0.1 M HCl (pH 2.0, adjusted with a HCl solution to 6 M) and, after its addition, samples underwent the second incubation step for 3 h at 37 °C under stirring conditions (150 rpm). Finally, the intestinal fluid was simulated by adding 280 mg of pancreatin and 1.75 mg of bile salts dissolved in 62.5 mL of 0.1 M NaHCO_3_ (pH 7.0, adjusted with a NaOH solution to 6 M) to the samples. At the end of the third incubation (3 h at 37 °C under stirring conditions (150 rpm)), the enzymatically digested samples were used as substrates for the fecal microbiota inoculum.

#### 2.3.2. In Vitro Colonic Fermentation of Digested *T. spicata*

**Figure 1:**
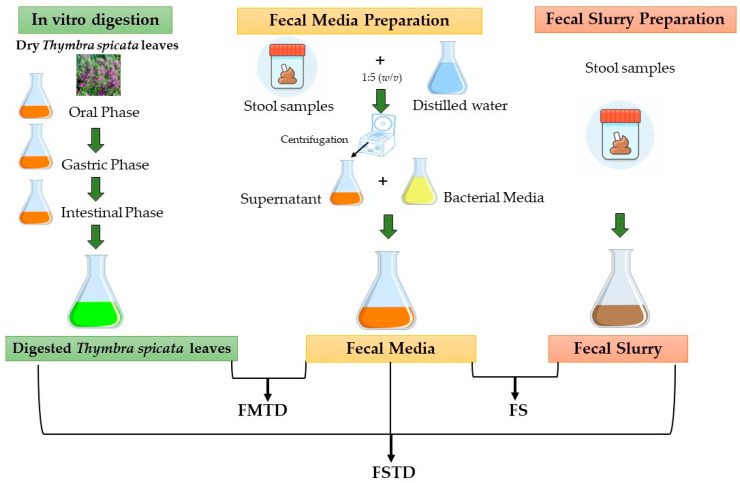
Representative scheme for the three samples obtained by in vitro simulation of gastrointestinal digestion and in vitro colonic fermentation. FMTD, fecal media and *T. spicata* digest; FS, fecal slurry; FSTD, fecal slurry and *T. spicata* digest.

As detailed by Vacca et al., the digested samples constituted a part of the fecal media. To obtain fecal media, a fecal pool containing equal stool samples from 3 healthy donors was added, in a ratio of 1:5 (*w*/*v*), to distilled water. The suspension, homogenized for 3 min with a lab stomacher in bags with a filter (250 μm), was centrifuged at 10,000× *g* for 10 min to recover supernatants. The previously digested *T. spicata* samples were added in a ratio of 1:4 (*v*/*v*) to supernatants and supplemented with K_2_HPO_4_•2 g/L, C_2_H_3_NaO_2_•5 g/L, C_6_H_17_N_3_O_7_•2 g/L, MgSO_4_•0.2 g/L, MnSO_4_•0.05 g/L, glucose•2 g/L, inulin•4 g/L, fructo-oligosaccharides•4 g/L and Tween 80 polysorbate•1 mL/L, then sterilized at 121 °C for 20 min. A cold-sterilized cysteine HCl (0.5 g/L), haemin (0.02 g/L), and vitamin K1 (10 µL/L) solution was finally added to form three different fecal media. Each fecal medium was inoculated with a fecal slurry (32% *w*/*v*) obtained by processing fresh feces from one healthy donor collected within 1 h. The simulated colonic fermentation in fecal batches (i.e., fecal medium with fecal slurry inoculum) accounted for a single-step anaerobic 42 h incubation at 37 °C under stirring conditions (150 rpm). Three final samples (namely, fecal media and *T. spicata* digest (FMTD); fecal slurry (FS); and fecal slurry and *T. spicata* digest (FSTD)) were obtained as depicted in Figure 1.

### 2.4. Fecal Microbiota Characterization

#### 2.4.1. Enumeration of Cultivable Microorganisms

Aliquots (5 g) from each fecal batch were added to 45 mL of sterile NaCl (0.9% *w*/*v*) solution and a serial 10-fold dilution was carried out subsequently. To inspect the viable microbiota, Plate Count Agar (*PCA*), Wilkins–Chalgren anaerobe agar (*WCAn*), de Man, Rogosa and Sharpe (*MRS*) agar, M17 agar, Violet Red Bile Glucose Agar (*VRBGA*) and modified Bifidobacterium agar (*mBifA*) were used as culture media for counts of total aerobes microbial (TAMC), total anaerobes microbial (TANMC), lactic acid bacteria (LAB), lactococci/streptococci, total coliforms and fecal *Bifidobacterium*, respectively. An exception was made for *mBifA*, which was purchased from Becton Dickinson GmbH (Heidelberg; Germany); other than this, all other media were purchased from Oxoid Ltd. (Basingstoke, Hampshire, England). *WCAn* and *mBifA* were anaerobically incubated, while the other media were incubated aerobically. The time and temperature of incubation followed those defined by the related manufacturer.

#### 2.4.2. DNA Extraction

Total DNA was extracted from stool samples. An aliquot (500 µL) of each sample was used for total DNA extraction. Samples were diluted in 1 mL of PBS-EDTA (phosphate buffer 0.01 M, pH 7.2, 0.01 M EDTA) and centrifuged (14,000× *g* at 4 °C for 5 min). Each pellet was washed twice. The extraction was performed using the FastDNA^®^ Pro Soil-Direct Kit (MP Biomedicals, Irvine, CA, USA). The quality check of the final DNA was carried out by spectrophotometric measurement at 260, 280, and 230 nm using a NanoDrop^®^ ND-1000 Spectrophotometer (ThermoFisher Scientific Inc., Milan, Italy).

#### 2.4.3. Real Time PCR

Fecal bacteria were quantified by Real Time PCR (qPCR). The primers used for microbial investigation are listed and described in Table 1. qPCR reactions were carried out on an Applied Biosystems 7300 Real-Time PCR System (Waltham, MA, USA). The total reaction mix (25 μL) contained 12.5 μL of SYBR Green Mix (# 1725271, Bio-Rad Laboratories S.r.l., Milano, Italy), 0.1 μL of 0.2 μM of primer, 11.4 μL of DNase and RNase-free water, and 1 μL of template. Each reaction was performed in triplicate. The amplification program consisted of 1 cycle of 95 °C for 3 min, followed by 40 cycles of 95 °C for 5 s, an appropriate annealing temperature (Table 1) for 30 s and 72 °C for 35 s. The PCR amplicon melting curve analysis started at a temperature of 60 °C and increased at 1 °C/5 s until reaching the final temperature of 95 °C.

Preliminary qPCR results have been reported in Log (Copy Number). The Copy Numbers (CN) and the logarithms were calculated based on the DNA concentration and amplicon length.

#### 2.4.4. Fecal Batch Volatile Organic Compound (VOC) Analysis

For the analysis of volatile organic compounds (VOCs), 2 mL each of the fecal medium and *T. spicata* digest, fecal slurry and fecal slurry, and *T. spicata* digest samples was taken and placed inside 20 mL vials that were closed with a magnetic cap with polytetrafluoroethylene septa (PTFE)/silicone. As internal standard, 10 µL of 4-methyl-2-pentanol was added. A Clarus 680 gas chromatograph (Perkin Elmer, Beaconsfield, UK) was used for GC-MS analysis. For the extraction of volatile compounds, a CombiPAL injector sample system (CTC Analytucs, Zwingen, Switzerland) and a 50/30 µm DVB/CAR/PDMS (Supelco, Bellefonte, PA, USA) were used. Helium was used as a carrier gas with a flow rate of 1 mL/min. Firstly, the samples were equilibrated for 15 min at 45 °C. To extract the volatile compounds, the fiber was exposed to 45 °C for 45 min in the headspace of the vials. The injection took place by heating the fiber inside the heated injection port to 220 °C in splitless mode. An Rtx-WAX column 30 m × 0.25 mm × 0.25 µm (Restek, Bellafonte, PA, USA) and coupled Clarus SQ8MS (Perkin Elmer) were used for VOCs separation with the source set at a temperature of 250 °C and the mass transfer line MS at a temperature of 230 °C. The oven temperature was initially 35 °C for 8 min and then increased to 60 °C with an increase of 4 °C/min, 160 °C with an increase of 6 °C/min and finally a temperature of 200 °C with an increase of 20 °C/min, at which it was maintained for 15 min. Electron ionization masses were recorded at 70 eV in the mass-to-charge ratio range from m/Z 34 to 350. For peak identification, chromatograms were analyzed using the NIST/EPA/NIH Mass Spectral Library with Search Program—Data ver. 2.3. Gaithersburg, MD, USA: National Institute of Standards and Technology (NIST); 2017) library. A peak area threshold of >1,000,000 and a probability of match of 85% or greater was used to identify the compounds. The relative concentration (expressed in µg/g of 4-methyl-2-pentanol) was estimated by the interpolation of the relative area with the air of the internal standard. The absolute concentrations of short chain fatty acids (SCFAs) and CVL (main phenolic and volatile compounds in *T. spicata*) in the samples were detected using standard curves.

### 2.5. Total Phenol Quantification

The total phenol content (TPC) was determined for each extract using the Folin–Ciocalteu method. Briefly, 100 μL aliquots of each sample were incubated with 0.5 mL of 10% (*w*/*v*) Folin–Ciocalteau reagent. After 5 min, 1.5 mL of Na_2_CO_3_ (2% *w*/*v*) was added and incubated in the dark at room temperature for 30 min. The absorbance was measured at 760 nm using a UV–Vis spectrophotometer Ultrospec 300 against a blank. The results were derived from a calibration curve of gallic acid (0–250 μg/mL) prepared from a stock solution and expressed in Gallic Acid Equivalents (GAE) per mL of extract.

### 2.6. Total Flavonoid Quantification

Total flavonoid content (TFC) was determined for each extract using aluminum chloride (AlCl_3_) colorimetric method. Briefly, 1 mL aliquot of each sample was mixed with 0.2 mL of 10% (*w*/*v*) methanolic AlCl_3_ solution, 0.2 mL (1 M) potassium acetate and 5.6 mL distilled water. After = incubation at room temperature in the dark for 30 min, the absorbance was measured at 415 nm using a UV–Vis spectrophotometer Ultrospec 300. The results were derived from a calibration curve of quercetin (0–200 μg/mL) prepared from a stock solution. The results have been expressed in mg of Quercetin Equivalent (QE) per mL of extract.

### 2.7. DPPH Radical Scavenging Assay

The radical scavenging activity of each extract was measured using the 1,1-diphenyl-2-picrylhydrazyl (DPPH) method. Briefly, from each sample, a 100 µL serial dilution was prepared to the following concentrations: 100%, 50%, 25%, 12.5%, and 6.25%. Then, in a 96-multiwell plate, 50 μL of each sample were added to 200 μL of DPPH (0.1 mM in methanol) solution. The mixtures were incubated at 37 °C in darkness for 20 min, and then the absorbance was measured at 490 nm using a microplate reader UV–Vis spectrophotometer Ultrospec 300 against an equal amount of DPPH solution as a blank. The percentage of DPPH scavenging was estimated using the equation:

% scavenging activity = [(Abs control − Abs sample)]/(Abs control)] × 100

### 2.8. Cell Culture

Caco-2 cells were grown and maintained in DMEM GlutaMAX supplemented with 10% fetal bovine serum, 100 i.u./mL penicillin, 100 μg/mL streptomycin and 1% non-essential amino acids, at 37 °C in a humidified atmosphere containing 5% CO_2_.

For the Ussing chamber experiments, Caco-2 cells were grown on Snapwell™ (Corning Incorporated, Corning, NY, USA) supports with a 1.12 cm^2^ cell growth area (#3801); the cells were seeded at a density of 2.2 × 10^5^ cells/cm^2^ on filters.

### 2.9. Ussing Chamber Studies

Transepithelial measurements were performed on Caco-2 cells grown as monolayers for 20 days. Inserts were mounted into Easy Mount Ussing chambers (Physiologic Instruments Inc., Reno, NV, USA).

Apical and basolateral hemi-chambers were filled with 5 mL of Ringer’s solutions containing (in mM): 115 NaCl, 25 NaHCO_3_, 0.4 KH_2_PO_4_, 2.4 K_2_HPO_4_, 1.2 MgCl_2_, 1.2 CaCl_2_, and 10 glucose (pH 7.4, 300 mOsm). The solution was gassed with a 95% O_2_ and 5% CO_2_ mixture and constantly recirculated by gas bubble lifting.

The short circuit current (Isc) and the transepithelial resistance (Rt) were measured as previously described [31].

### 2.10. Statistical Analyses

As appropriate, data were expressed as means with standard error of the mean (SEM). For continuous variables, comparisons were undertaken using the two-tailed *t*-test, Tukey’s test, or Mann–Whitney U test corrected for multiple comparisons (Dunn’s statistics). The analysis of variance was carried out by Two-way ANOVA testing. For all comparisons, differences were considered as significant when reaching a *p*-value (*p*) < 0.05. Subsequently, for normalization (Z-score), multivariate analyses were carried out by running a principal component analysis (PCA) or hierarchical clustering (based on Ward’s metrics and Euclidean distance) in the R-environment by running the “FactoMineR” package (Multivariate Exploratory Data Analysis and Data Mining) version 2.4, available in the CRAN repository. A volcano plot combining results from the Fold Change (FC) analysis and nonparametric Wilcoxon rank-sum test was used to intuitively select significant VOCs based on either biological significance, statistical significance, or both. Statistical analyses and a graph visualization of the electrophysiological data were performed using GraphPad Prism version 8.0 (GraphPad Software, San Diego, CA, USA). 

## 3. Results 

### 3.1. Chemical and Nutritional Value of Thymbra spicata

Leaves of *T. spicata* were fully characterized, as depicted in Table 2. The data refer to macronutrients, minerals (ash), and energy content.

More than 50% of plant materials were carbohydrates (52.7 g per 100 g of the *T. spicata* leaves). Total dietary fiber was measured at 12.3 g/100 g, and the sum of the simple sugars (listed by specific sugar types, along with their values in g per 100 g) was 1.74 g. Total protein and fat content were 6.19 and 4.3 g/100 g, respectively. The contents of saturated fatty acids were low (0.9 g), while unsaturated fatty acids were more represented. Oleic acid (C18:1) and linoleic acid (C18:2) accounted for 40.6% and 30.5% of fat content, respectively. Other constituents, such as moisture and ash, were present in moderate amounts (17.0 and 9.11 g/100 g, respectively). Finally, the energy value of *T. spicata* leaves was 324 kcal/100 g, which is equal to 1367 kJ/100 g.

### 3.2. Effect of T. spicata on Fecal Microbiota after Simulated Digestion and Colonic Fermentation

In vitro simulated digestion was performed to inspect the physiological effects of *T. spicata* on the viable cell density of the main cultivable bacterial groups belonging to the intestinal microbiota. The differences observed were determined through the simulated digestion of *T. spicata*, comparing it against the same medium and culturing conditions without the presence of the plant’s macro and micronutrients. While a similar trend characterized the experiments, indicating a decreasing density from FS to FSTD, no significant differences were detected based on the presence of *T. spicata* in the medium. In fact, the densities of both total aerobes (~9 logs CFU/mL) and anaerobes (~8 logs) were comparable between FS and FSTD samples (Table 3). Consistently with these findings, even *Lactococci* and *Bifidobacterial* cells did not exhibit substantial variations. Although not statistically significant (*p* > 0.05), lactobacilli and *Enterobacteriaceae* showed a ~0.5 log higher density in FS compared to FSTD. The most notable difference was observed in the density of *Enterocc*i, reaching approximately 5 logs in FS, while reporting mean values of 3.5 logs in FSTD. *Clostrid*i, on the other hand, demonstrated the lowest *p*-value (*p* = 0.09) among the sample comparisons, with a higher density in FS (~8.8 logs) compared to FSTD (~8.3 logs).

**Figure 2:**
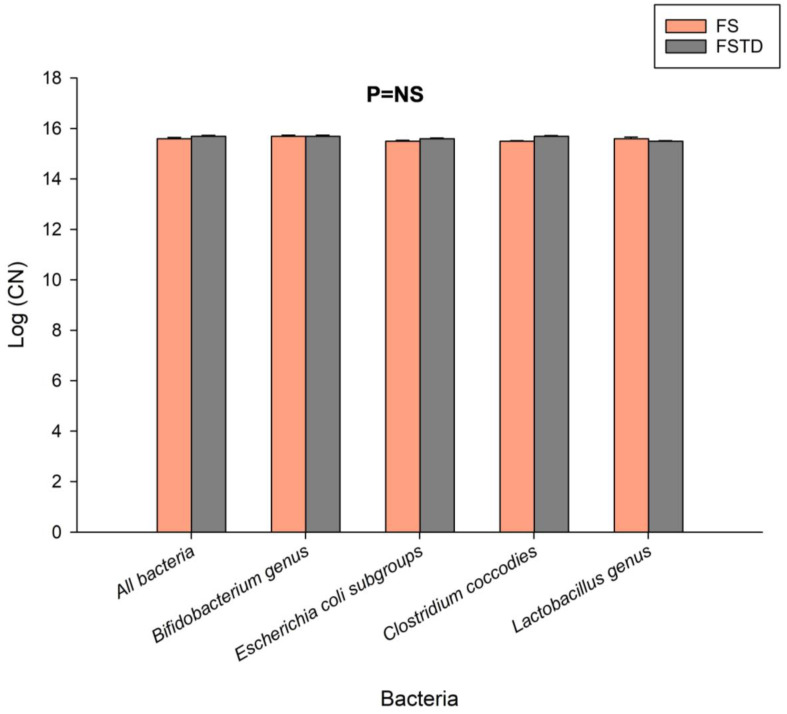
Log (Copy Number) based on DNA concentration and amplicon length after a qPCR of extracted DNA derived from the batch fermentation of FS and FSTD. Tested bacteria were: all bacteria, *Bifidobacterium genus*, *Escherichia coli* subgroups, *Clostridium coccodies*, and *Lactobacillus genus*. *p*-value obtained by Student’s *t*-test.

In addition, to better inspect the effects of *T. spicata* on microbiota composition in the fecal batch, a panel of microbial genera and species was investigated by qPCR analysis (Figure 2). In line with the viable microbial cells count, the Log (Copy Number) values for all tested bacteria, which were calculated based on the DNA concentration and amplicon length, were comparable between FS and FSTD.

### 3.3. Effects of T. spicata on Fecal Volatile Organic Compounds (VOCs)

**Figure 3:**
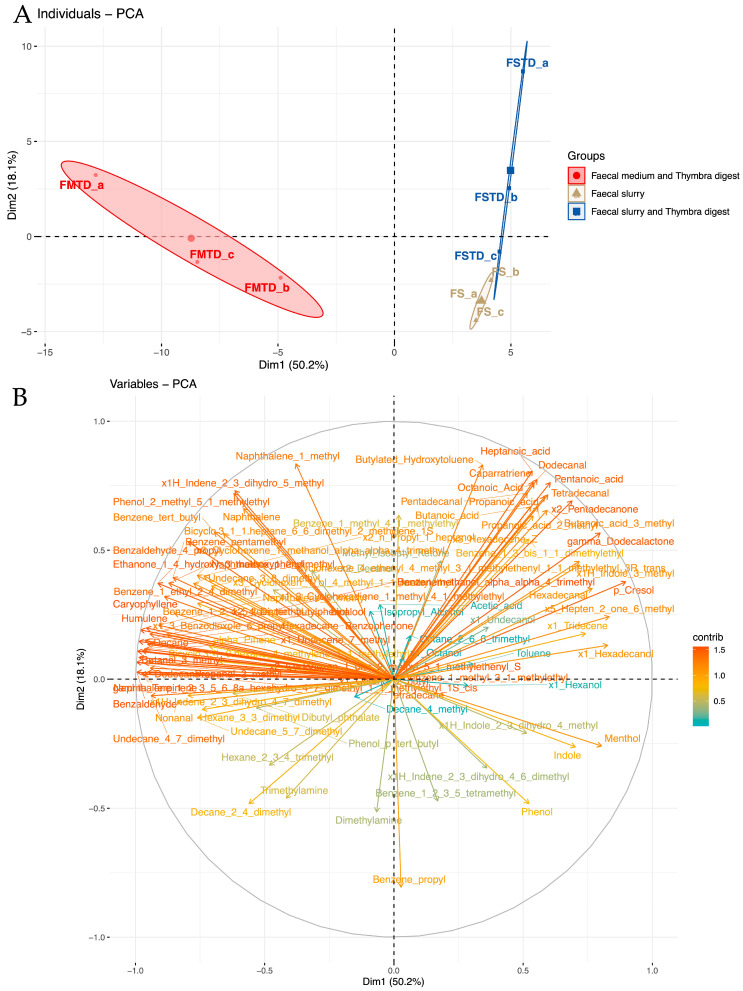
Principal component analysis (PCA) of volatile organic compounds (VOCs) in fecal medium and *T. spicata* digest (FMTD), fecal slurry (FS) and fecal slurry and *T. spicata* digest (FSTD) samples. The distributions of sample replicates (a,b,c) were plotted according to the first two components Dim 1 (50.2%) and Dim 2 (18.1.%) (**A**), and the contributions of each variable are shown in the loading plot (**B**).

With the aim of ascertaining the impact of *T. spicata* on the metabolic activities of the main cultivable bacterial group from the gut microbiota, we performed HS-SPME GC-MS analyses to evaluate the VOCs. For this purpose, the volatile profiles were evaluated in the fecal medium and *T. spicata* digest (FMTD), fecal slurry (FS) and fecal slurry and *T. spicata* digest (FSTD) groups (Supplementary Table S1). In total, 94 VOCs were identified and divided into the following chemical classes: alcohols (n = 8), carboxylic acids (n = 9), hydrocarbons (n = 23), indoles (n = 3), phenols (n = 5) and terpenes (n = 18), plus another 28 compounds not belonging to any of the classes previously mentioned. In addition, the results obtained were analyzed by multivariate analysis of the principal component (PCA). The unsupervised PCA, which explained 68.3% of the variance, showed that there was a difference distribution among samples containing fecal slurry and samples with only fecal medium (Figure 3).

**Figure 4:**
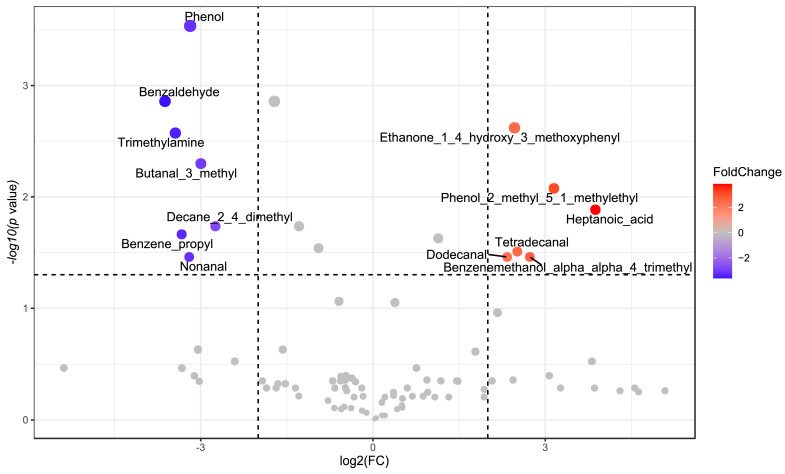
Volcano plot of statistically significant VOCs in fecal slurry. Increases (red) and decreases (blue) in VOC concentration in FSTD compared to FS. Non-statistically significant differences of VOCs are reported as grey dots. Wilcoxon rank-sum tests (*p* < 0.05) combined with a fold change analysis (log2FC > 2) were used.

The analysis has shown that the FMTD samples were placed in the first and fourth quadrants, while samples of fecal slurry with and without the addition of *T. spicata* digest were placed in the second and third quadrants. To evaluate the statistical differences of the VOCs between those of FS and FSTD, a paired comparison was undertaken through the Wilcoxon rank–sum test and a fold change analysis (log2FC > 2) (Figure 4).

As shown in the Volcano plot, the addition of *T. spicata* led to increases in tetradecanal; dodecanal; heptanoic acid; ethanone, 1-(3-hydroxy-4-methoxyphenyl)-; phenol, 2-methyl-5-(1-methylethyl)- and benzenemethanol, alpha,alpha,4-trimethyl-, and to decreases in phenol; nonanal; decane,2,4-dimethyl-; benzene, propyl-; butanal, 3-methyl-; trimethylamine and benzaldehyde.

The concentrations of SCFAs and CVL (the main volatile phenolic compound in *T. spicata*) were also evaluated. As depicted in Table 4, higher concentrations of propanoic acid, butanoic acid, and isovaleric acid were observed in FSTD compared to FS, while in the FMTD, as expected, SCFAs were not detected.

### 3.4. Effects of Microbial Fermentation on T. spicata Phenolic Content and Radical Scavenging Activity

**Figure 5:**
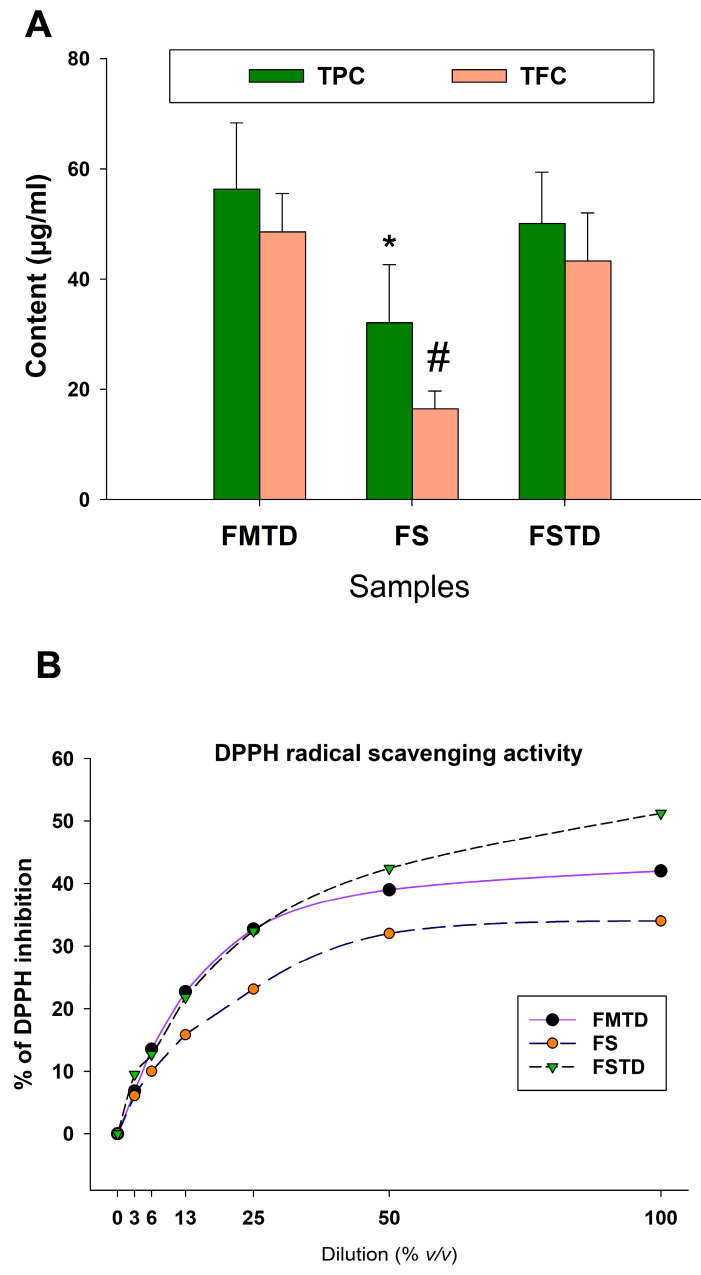
(**A**) Total phenol (TPC) and flavonoid (TFC) contents measured in FS, FMTD and FSTD. TFC is expressed as µg Gallic Acid Equivalent GAE per mL of sample. TFC is expressed as µg Quercetin Equivalent QE per mL of sample. * indicates difference compared to another sample (*p* < 0.05) for TPC; # is difference compared to another sample (*p* < 0.05) for TFC. (**B**) Dose-dependent DPPH radical scavenging activity expressed as a percentage of radical inhibition.

The possible modification of the phenolic content, flavonoid content, and antioxidant capacity of *T. spicata* after its interaction with human intestinal microbiota was investigated by colorimetric assays on FS, FMTD, and FSTD. As shown in Figure 5A, the TPC and TFC in FS were lower (32.1 ± 5.4 and 16.5 ± 3.7, respectively) than in FMDT and FSDT (138.9 ± 22.5 vs. 139.4 ± 27.3 µgGAE/mL for TPC and 43.3 ± 5.8 vs. 48.6 ± 8.3 µgQE/mL for TFC, respectively).

The antioxidant capacity was tested by a free-radical scavenging ability (DPPH) assay. The dose-dependent DPPH inhibition (%) is represented in Figure 5B. In line with TPC and TFC, the maximum percentage of DPPH inhibition was observed in TE, and the lowest percentage was seen in FS. FMTD and FSTD showed similar trends of radical scavenging with slightly higher activity observed in FSTD compared to FMTD at higher concentrations (5 mg/mL).

**Figure 6:**
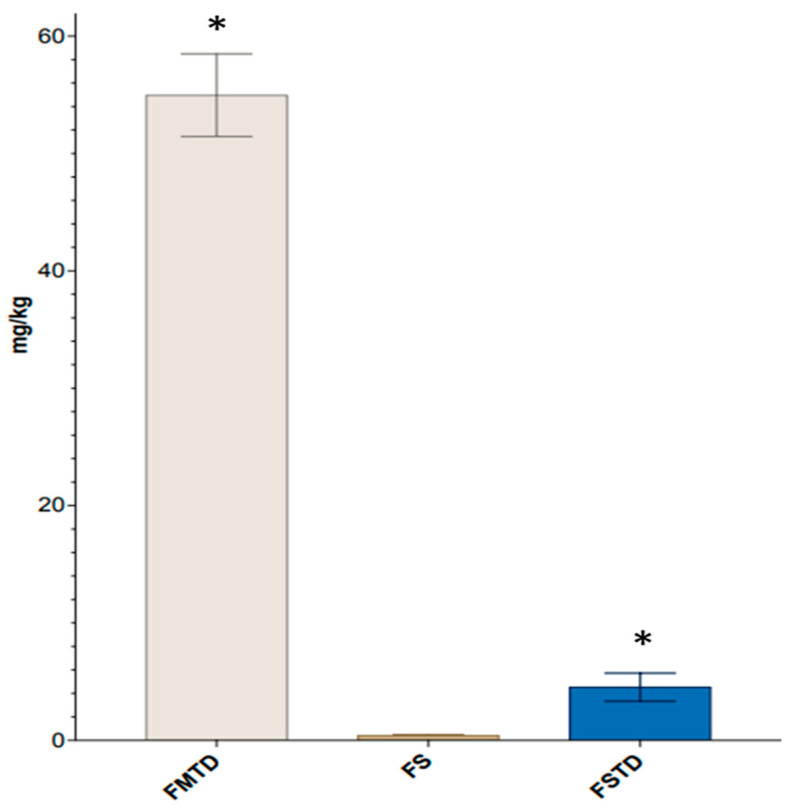
Concentration (mg/kg) of carvacrol determined by GC-MS in fecal medium and *T. spicata* digest (FMTD), fecal slurry (FS) and fecal slurry and *T. spicata* digest (FSTD) samples. Statistical differences were evaluated by One-way ANOVA followed by Tukey’s test. Asterisk (*) indicates statistically significant differences (*p*-value < 0.05).

The content of CVL was calculated in all samples (Figure 6). The highest CVL concentration was found in FMTD (54.96 mg/kg). The CVL concentration was dramatically decreased to 5 mg/kg in FSTD, while the CVL concentration in FS was 0.43 mg/kg. 

### 3.5. Effects of T. spicata Ethanolic Extract and Carvacrol on the Intestinal Barrier Function

To evaluate the effect of *T. spicata* ethanolic extract (TE) on the intestinal barrier function, the electrophysiological properties of Caco-2 monolayers were investigated using the Ussing chamber technique. This technique monitors concurrent changes in short-circuit current (Isc), a measure of net electrogenic transepithelial ion flux, as well as transepithelial resistance (Rt), as a measure of barrier integrity.

**Figure 7:**
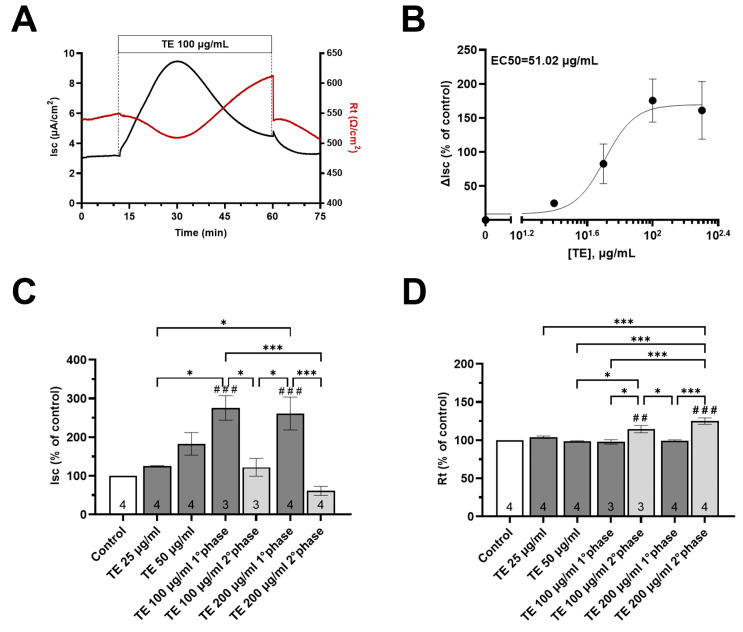
Effect of apical addition of *T. spicata* ethanolic extract on Caco-2 monolayers. (**A**) Representative traces of short circuit current (Isc) and transepithelial resistance (Rt) after *T. spicata* ethanolic extract (TE) addition (100 µg/mL). (**B**) Dose–response curve of *T. spicata* ethanolic extract-evoked Isc changes. The plots show the means (represented by black dots) ± the SEM of Isc measured at each concentration. The half-maximal inhibition constant (EC_50_) for TE was 51.02 µg/m. (**C**,**D**) Changes in the electrophysiological parameters induced by apical TE addition. Results are expressed as percentages of the Isc and Rt basal values. Sample size is represented by the number at the bottom of each bar. Values are reported as mean values ± SEM, with the control represented by a white bar and TE addition at concentrations of 25, 50, 100, and 200 µg/ml depicted by light gray and dark gray bars for the first and second phases, respectively. ## *p* < 0.01, ### *p* < 0.001 vs. basal condition calculated by one-way analysis of variance (ANOVA) with Dunnett’s multiple comparison test; * *p* < 0.05, *** *p* < 0.001 values calculated by one-way analysis of variance (ANOVA) with Sidak’s multiple comparison test, indicating how much the results differ from those of other treatments.

Following an equilibration period of 15 min, the baseline values of short-circuit current (Isc) and transepithelial resistance (Rt) were 2.94 ± 0.23 μA/cm^2^ and 517 ± 31.8 Ω/cm^2^, respectively (n = 37). The addition of increasing concentrations of TE at the apical side of Caco-2 monolayers increased the Isc when applied at 25 and 50 µg/mL (+24.8% and +82.7% compared to baseline, respectively), without affecting the Rt (Figure 7B–D).

Of note, higher concentrations of TE (100 and 200 µg/mL) evoked biphasic responses, with a significant and transient increase in the Isc (+175.6% and 161.1%) not associated with changes in the Rt (−2.21 and −0.72%), followed by a slight increase in the Isc at 100 µg/mL (+22.1%) and a decrease at 200 µg/mL (−38.7%) accompanied by a significant increase in the Rt (+14.5% and +25.1%) (Figure 7A,C,D).

The dose–response curve of TE was characterized by measuring the percentage change in short-circuit current with increasing concentrations of TE. The EC_50_ value, representing the concentration at which Isc exhibits a 50% change from baseline, was determined to be 51.02 µg/mL (Figure 7B).

**Figure 8:**
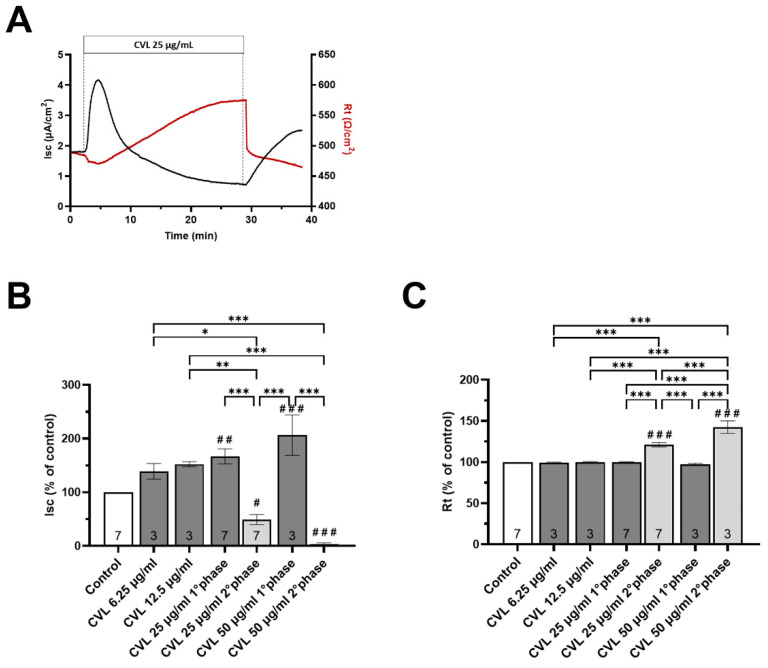
Effect of apical addition of Carvacrol on Caco-2 monolayers. (**A**) Representative traces of short circuit current (Isc) and transepithelial resistance (Rt) after carvacrol (CVL) addition (25 µg/mL). (**B**,**C**) Changes in the electrophysiological parameters induced by apical CVL addition. Results are expressed as percentages of the Isc and Rt basal values. Sample size is represented by the number at the bottom of each bar. Values are reported as mean values ± SEM, with the control represented by a white bar and TE addition at concentrations of 25, 50, 100, and 200 µg/ml depicted by light gray and dark gray bars for the first and second phases, respectively. # *p* < 0.05, ## *p* < 0.01, ### *p* < 0.001 vs. basal condition calculated by one-way analysis of variance (ANOVA) with Dunnett’s multiple comparison test; * *p* <0.05, ** *p* < 0.01, *** *p* < 0.001 values calculated by one-way analysis of variance (ANOVA) with Sidak’s multiple comparisons test, indicating how much the results differ from those of other treatments.

Given that CVL is the major component of TE (constituting about 25% of TE [15]), our specific aim was to determine whether the response observed after the addition of TE could be attributed to the presence of CVL. To address this, we investigated the effect of CVL by using 6.25, 12.5, and 25 and 50 µg/mL, which correspond to the amounts of CVL contained in the different doses of the ethanolic extract tested. As illustrated in Figure 8B,C, the application of increasing concentrations of CVL at the apical side resulted in an increase in Isc (+39% and +52,4% for 6.25 and 12.5 µg/mL, respectively) without any change in the Rt. However, when a higher concentration of CVL (25 and 50 µg/mL) was applied, a biphasic response similar to that evoked by 100 and 200 µg/mL TE was observed. In fact, the presence of CVL elicited a significant increase in the Isc (+66.9% and 106.3%) with no change in the Rt, followed by an Isc reduction (−50.8% and −97.1%) with a significant increase in Rt (+21% and +42.5%). The similarities in the TE and CVL responses are very intriguing since we could speculate that CVL plays a key role in modulating the electrical properties of intestinal cells.

### 3.6. In Vitro Digestion and Microbial Fermentation Influences the Effect of T. spicata on the Intestinal Barrier

**Figure 9:**
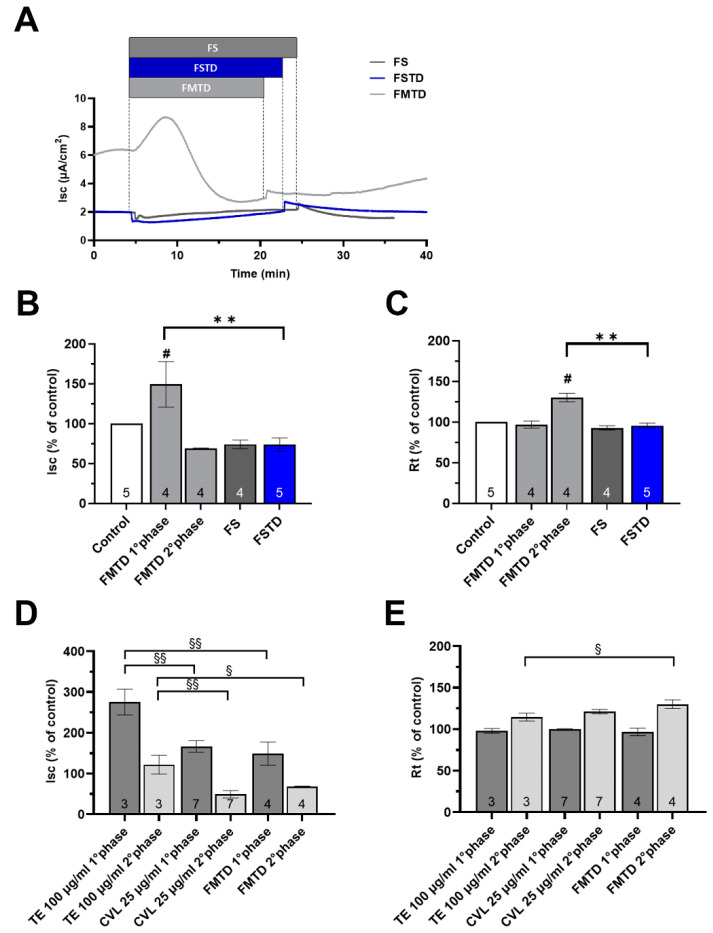
Effects of apical addition of fecal medium and T. spicata digest (FMTD), fecal slurry (FS), and fecal slurry and T. spicata digest (FSTD) on Caco2 monolayers. (**A**) Representative trace of short circuit current (Isc) after FMTD, FS and FSTD addition. (**B**,**C**) Changes in the electrophysiological parameters induced by apical FMTD, FS and FSTD addition. (**D**,**E**) Changes in the electrophysiological parameters induced by apical *T. spicata* ethanolic extract, carvacrol and FMTD addition. Results are expressed as percentages of the Isc and Rt basal values. Sample size is represented by the number at the bottom of each bar. Values are reported as mean values ± SEM, with the control represented by a white bar and TE addition at concentrations of 25, 50, 100, and 200 µg/ml depicted by light gray and dark gray bars for the first and second phases, respectively. # *p* < 0.05 vs. basal condition calculated by one-way analysis of variance (ANOVA) with Dunnett’s multiple comparison test; ** *p* < 0.001 vs. FSTD condition calculated by Bonferroni’s multiple comparisons test; § < 0.05, §§ *p* < 0.01 values calculated by one-way analysis of variance (ANOVA) with Bonferroni’s multiple comparisons test, indicating how much the results differ from those of other treatments.

To study the role of the gut microbiome on the effect of *T. spicata* on Caco-2 monolayers, we examined the effects of FMTD, FS, and FSTD. These series of experiments were performed using a concentration of 100 µg/mL, which, as described previously, induced the maximal cell response. As shown in Figure 9A,B, the addition of FMTD elicited a response characterized by two phases. Initially there was a substantial and statistically significant increase in the Isc (+49%) and no change in the Rt. Subsequently, in the second phase, the I_SC_ decreased and the Rt increased (−31.2% and +30% compared to the basal value, respectively). Interestingly, the apical application of FS or FSTD did not affect the electrical properties of the Caco-2 monolayers since neither the Isc nor the Rt were significantly altered. Moreover, the Isc responses evoked by FMTD were significantly lower than those observed for TE, but did not differ from the response to CVL.

## 4. Discussion

The MD incorporates a wide variety of plant-based foods, including fruits, vegetables, whole grains, nuts, seeds, and herbs [32]. Besides its richness in essential nutrients, fibers, and vitamins, the components of the MD are rich in polyphenols. The potential effects of these substances are mainly dependent on their interaction with the gut barrier, where polyphenols may contribute to the modulation of gut microbiota by increasing the beneficial bacteria and decreasing the overgrowth of pathogenic bacteria. On the other hand, the gut microbiota may bio-transform polyphenols to increase their bioavailability [33]. Understanding the physiological interactions of components/substances in the MD with gut microbiota and gut barrier is of key importance and has attracted the attention of researchers worldwide. 

*Thymbra spicata* is rich in polyphenols and essential oils with a long-standing history and tradition of use in the Eastern Mediterranean region for medical and foods purposes. *Thymbra spicata* is a thyme-like plant belonging to the *Lamiaceae* family [12]. Recently, *T. spicata* has gained much popularity as a remedy to combat different metabolic disorders [34]. A protective role of *T. spicata* was also reported as it was used as an anti-genotoxic [35] and anti-coagulant agent [36]. However, aspects including the nutritional value and the physiological interaction between the ingested *T. spicata* and the gut have still not been identified. In this preliminary study, we assessed, for the first time, the nutritional and caloric profiles of *T. spicata* leaves. These aspects may give important insight into the nutritional and bio-functional characteristics of *T. spicata*, helping us to better understand its physiological interaction with gut microbiota and the intestinal barrier, which were assessed by well-established in vitro models. *T. spicata* contains a considerable amount of fiber and unsaturated fatty acids, as well as CVL (polyphenols). In line with its high fiber content, we found an increase in the production of SCFAs after microbial fermentation.

A growing body of scientific research supports the potential health benefits of dietary polyphenols on the gastrointestinal tract and the gut microbiota. Plant-based polyphenols undergo metabolization and biotransformation during gastrointestinal digestion, and through their interaction with the gut microbiota. Polyphenols and their metabolites then exert several effects on the human gastrointestinal tract, including the modulation of gut microbiota, the improvement of the intestinal barrier, and a reduction in inflammation and oxidative stress [37].

We previously reported the possible antisteatotic and antioxidant effects of *T. spicata* on the in vitro hepatic steatosis model, whereby we proved that *T. spicata* ameliorated hepatic steatosis by reducing lipid droplet numbers and sizes, intracellular ROS production, and lipid peroxidation [12]. CVL, a monoterpene phenolic compound, is the main compound of *T. spicata* essential oils and alcoholic extracts. Previously, we reported an antisteatotic effect, as well as the albumin-binding properties in a high-fat environment, of CVL using in vitro models [27]. In addition, we have recently reported possible patterns of phenolic compounds in *T. spicata* and their bioactivity during gastrointestinal transit via a simulated in vitro gastrointestinal (GI) digestion of *T. spicata* [28]. The simulated GI digestion reduced the phytochemical contents in *T. spicata* extracts but improved their antioxidant potential. 

Given their beneficial effects, *T. spicata* and CVL were additionally investigated in this study in term of their physiological interaction with gut barriers. We incubated the digested *T. spicata* with a fecal microbiota fermentation model to understand the possible effects of *T. spicata* on the growth of human microbiota. *T. spicata* tended to reduce bacterial growth. Due to its richness in essential oils, especially CVL, *T. spicata* might exert an antimicrobial effect [17,18,19,38]. This could be useful for the management of pathogenic bacterial overgrowth during dysbiosis, which may occur under different disease conditions. Several studies using cellular or animal models have described the modulatory effects of CVL and CVL-rich essential oils (EOs) on gut microbiota [11]. EOs rich in CVL increased the relative abundance of some beneficial species such as *Bacilli*, *Lactobacillales*, and *Veillonellaceae* in weaned piglets [39], and improved microbiota composition, as well as piglet health and performance [40]. Additionally, CVL increased the relative abundance of *Lactobacillus* spp. in the chicken gut [41]. CVL supplementation also improved gut dysbiosis by enhancing the abundance of beneficial bacteria such as *Firmicutes*, and reducing the abundance of harmful bacteria such as *Proteobacteria* [42]. The treatment of germ-free zebrafish colonized by microbiota with CVL-rich EOs exerted an anti-inflammatory effect by reducing serum amyloid and interleukin 1β, 8, and upregulated the expression of tight junction proteins such as claudin-1 and occludin-2 [43]. In intestinal cells, i.e., Caco-2 [44], Vero cells [45], and IPEC-J2 [46], CVL treatment resulted in a significant reduction of bacterial pathogenicity and toxicity in vitro. One additional aspect to consider is the possibility that the changes to the gut microbiota induced by *T. spicata* can have beneficial effects on the gut bile acid pool and their overall effects as anti-inflammatory agents, and can act agonists of ileal membrane-associated and nuclear receptors.

The effects of *T. spicata* on microbiota are supported by metabolomic analyses of VOCs. This analysis has allowed us to highlight the differences in microbiota metabolism, and facilitated the detection and quantification of metabolites upon *T. spicata* fermentation. The results indicate that *T. spicata* stimulated specific microbial pathways, resulting in the increased production of hepatonic acid, medium-chain fatty acids (MCFAs), and SCFAs (mainly propanoic acid). Beneficial effects of SCFAs are expected on the gut barrier, as well as at a systemic (metabolic) level. The increase in MCFAs and SCFAs can be beneficial in patients with irritable bowel syndrome, where MCFAs and SCFAs are reduced [47]. Besides this, the partial increase in SCFAs could be attributed to the high content of fiber in *T. spicata* leaves. The increase in other metabolites such as ethanone, 1-(3-hydroxy-4-methoxyphenyl)-, phenol, 2-methyl-5-(1-methylethyl)-, benzenemethanol and alpha,alpha,4-trimethyl- point to the modulation of microbial activities associated with phenolic metabolism and biotransformation. Notably, a decrease in trimethylamine is observed in the *T. spicata* metabolome, which could be of great relevance to cardiovascular health, as trimethylamine is a precursor of trimethylamine N-oxide (TMAO), which has been linked to cardiovascular risk [48].

Regarding the effects of *T. spicata* on the intestinal barrier function, ethanolic extract (TE) and CVL, as well as digested *T. spicata* with or without fecal fermentation samples, were tested. We found that the apical addition of the TE (100 and 200 µg/mL) and CVL (50 µg/mL) transiently increased the ionic permeability without affecting the integrity of Caco-2 monolayers, as suggested by the biphasic increase in short circuit current (Isc) and absence of changes in transepithelial resistance (Rt). Interestingly, both TE and CVL exhibited a biphasic response with a significant increase in intestinal transepithelial resistance observed in the second phase. The biphasic response observed in Caco-2 monolayers exposed to 25 µg/mL of CVL might be attributed to various molecular and cellular processes. The initial increase in short-circuit current could be linked to the activation of ion channels or transporters on the apical membrane of Caco-2 cells.

CVL, known for its modulatory effects on ion channels, may initially enhance the movement of ions across the cell membrane, resulting in an increased Isc. The subsequent reduction in Isc might be associated with cellular adaptation or regulatory mechanisms. It is possible that prolonged exposure to CVL triggers feedback mechanisms that downregulate ion transport, leading to a decrease in Isc. The observed biphasic response evoked by CVL shares similarities with the response induced by 100 µg/mL of *T. spicata* ethanolic extract. This is not surprising, since CVL is the most abundant component of the ethanolic extract, and therefore it is conceivable that it could play a key role in determining the electrical changes recorded in our studies. 

Both TE and CVL initially increased Isc, suggesting a commonality in their early effects on ion transport. The subsequent reduction in Isc in the second phase might involve shared or convergent signaling pathways.

The ability of the *T. spicata* to increase the ionic permeability and the transepithelial resistance was maintained after simulated gastrointestinal digestion (FMDT sample), although with lower efficacy due to the partial loss of phytochemical compounds during digestion, as reported by our previous study [28], and CVL, which was lower in the digested sample than the ethanolic extract. However, when pre-digested *T. spicata* were subjected to human microbial fermentation, mimicking the physiological interaction between digested food and healthy human gut microbiota, an effect on Caco-2 monolayers was not observed. This could be also explained by the dramatic reduction in CVL in the fermented sample. The results of this series of experiments demonstrate that although the fecal slurry (used as negative control) does not have any significant influence on intestinal function, it has a strong impact on the ability of *T. spicata* to modulate the permeability of the intestinal barrier, and its function, since the biological effects observed in non-fermented *T. spicata* (TE and FMDT) on the Isc and Rt were completely abolished. Taken together, we can observe a correlation between CVL and the effects on Caco-2 monolayers, and therefore, we could speculate that CVL plays a key role in determining the biological effects of *T. spicata.*

Although no studies have elucidated the effects of *T. spicata* on Caco-2 cells, a few studies have reported the beneficial effects of CVL. For example, CVL at lower concentrations exerted a cytoprotective and DNA-protection effect on free radical-injured Caco-2 cells [49,50]. The dual-phase response in Caco-2 monolayers exposed to FMTD at 100 µg/mL likely involves intricate interactions between *T. spicata* components and the gut, in terms of polyphenolic metabolites and digestion, leading to distinct cellular responses. This response differs from the effects seen with *T. spicata* ethanolic extract or CVL, indicating unique microbial–plant–metabolite interactions, supported by the different levels of polyphenols and polyphenol metabolites. 

The gut microbiome is known for its dynamic metabolic activities. Metabolites produced during fecal medium preparation may contain active metabolites (i.e., enzymes) that could interact with *T. spicata*, and which may play a role in modulating cellular responses. Furthermore, microbial signals could potentially interact with the cellular pathways involved in ion transport. To exclude such effects, we also tested the effects of fecal slurry (FS) on Caco-2 monolayers, and found no significant effect. 

Although no significant changes in the amount of polyphenols (TPC) or the radical scavenging activity (DPPH) were observed between FMTD and FSTD, the CVL content in FMTD was ten times higher than in FSTD. This crosstalk and the content of CVL may contribute to the changes observed in Isc. Unravelling these mechanisms will enhance our understanding of how *T. spicata*’s different interactions shape cellular responses in the gut epithelium.

*T. spicata* manifested an increase in intestinal barrier resistance under physiological conditions. This could be relevant to the further evaluation of the beneficial effects of *T. spicata* on the modulation of gut barrier integrity during conditions of “leaky gut”, where the intestine’s epithelial resistance is altered due to different states of epithelial damage. 

## 5. Conclusions

Research has shown that dietary polyphenols can positively influence the gastrointestinal tract and gut microbiota. *Thymbra spicata*, a Mediterranean plant rich in polyphenols, may play an important role in gut physiology. Here, we found that *T. spicata*’s nutritional and phytochemical composition, as well as its antioxidant potential, elevate its potential use as a valuable component of the diet. Furthermore, its interaction with the gut microbiota demonstrates its ability to help maintain a balanced microbial environment and beneficial metabolites, and it can potentially address pathogenic bacterial overgrowth during dysbiosis, a condition associated with various diseases. The ethanolic extract of *T. spicata* has shown promise for use in enhancing intestinal barrier resistance, which is crucial for preserving gut integrity and preventing conditions such as leaky gut syndrome.

In addition, understanding the alterations in microbial populations and their potential functional implications in response to *T. spicata* is a critical aspect that warrants further investigation. Further studies must better define the mechanisms underlying the beneficial effects of *T. spicata* on microbial growth and the ionic permeability and barrier function of the intestinal tissue.

## Figures and Tables

**Table 1:** List of primers and related features.

Target	Primers	Primer Sequence (5′–3′)	Gene	Product Size (bp)	T Annealing (°C)	Reference
All Bacteria	Uni331F	TCCTACGGGAGGCAGCAGT	16S rRNA	466	58	[29]
	Uni797R	GGACTACCAGGGTATCTATCCTGTT				
*Lactobacillus* genus	Lp-F	AAAATCATGCGTGCGGGTAC	16S rRNA	341	55	[29]
	Lp-R	ATGTTGCGTTGGCTTCGTCT				
*Bifidobacterium* genus	Bifid-F	CTCCTGGAAACGGGTGG	16S rRNA	550	55	[29]
	Bifid-R	GGTGTTCTTCCCGATATCTACA				
*Escherichia coli* subgroup (*E. coli*, *Hafnia alvei*, *Shigella* spp.)	Eco-F	GTTAATACCTTTGCTCATTGA	V3-V4 hypervariable regions	340	60	[30]
	Eco-R	ACCAGGGTATCTAATCCTGTT				
*Clostridium coccoides* group	Ccoc-F Ccoc-R	AAATGACGGTACCTGACTAA CTTTGAGTTTCATTCTTGCGAA	16S rRNA	440	50	[29]

**Table 2:** Analytical values of chemical composition and nutritional values of *Thymbra spicata* leaves.

Compounds	Quantity	Compounds	Quantity
Total fat (g/100 g)	4.3	Total carbohydrates (g/100 g)	52.7
Saturated fatty acids (g/100 g)	0.9	Total dietary fiber (g/100 g)	12.3
Fatty acid methyl esters (% of fat content)		Simple sugars (g/100 g)	
C12:0—Lauric acid	<0.1	Fructose	0.03
C14:0—Myristic acid	<0.1	Galactose	n.d
C16:0—Palmitic acid	17.5	Glucose	1.69
C16:1—Palmitoleic acid	<0.1	Lactose	n.d
C17:0—Heptadecanoic acid	<0.1	Maltose	n.d
C17:1—Heptadecenoic acid	<0.1	Sucrose	0.02
C18:0—Stearic acid	2.6	Sum sugars	1.74
C18:1—Oleic acid	40.6	Nitrogen (g/100 g)	0.99
C18:2—Linoleic acid	35.5	Total protein (g/100 g)	6.19
C20:0—Arachic acid	2.6	Moisture (g/100 g)	17.0
C18:3—Linolenic acid	0.5	Salt (g/100 g)	0.009
C20:1—Eicosenoco acid	<0.1	Ash (g/100 g)	9.11
C22:0—Beenic acid	<0.1	Energy value (Kcal/100 g)	324
C22:1—Erucic acid	<0.1	Energy value (KJ/100 g)	1367
C24:0—Lignoceric acid	<0.1		

Cx:y, number of carbon atoms:number of double covalent bonds; n.d, not determined.

**Table 3:** Viable microbes of fecal slurry (FS) and fecal slurry and *T. spicata* digest (FSTD).

Viale Microbes (Log UFC/mL)	FS (Control)	FSTD	*p*-Value
Total aerobes	9.3 ± 0.3	9.0 ± 0.4	0.61
Total anaerobes	8.0 ± 0.5	8.0 ± 0.4	0.99
*Lactobacilli*	5.0 ± 1.0	4.4 ± 1.0	0.69
*Lactococci*	7.8 ± 0.4	7.6 ± 0.5	0.81
*Bifiobacteria*	7.1 ± 0.7	6.9 ± 0.5	0.86
*Enterobacteriaceae*	8.8 ± 0.2	8.2 ± 0.6	0.39
*Enterococci*	4.9 ± 0.1	3.5 ± 1.4	0.46
*Clostridi*	8.8 ± 0.1	8.3 ± 0.2	0.09

Data are expressed as Mean ± SEM; *p*-value obtained by Student’s *t*-test.

**Table 4:** Concentrations (ppm) of short-chain fatty acids in headspace of fecal medium and *T. spicata* digest (FMTD), fecal slurry (FS) and fecal slurry and *T. spicata* digest (FSTD) samples.

SCFAs (ppm)	FMTD	FS	FSTD
Acetic acid	n.d.	10.7 ± 2.3	10.1 ± 1.2
Propanoic acid	n.d.	4.8 ± 2.1 *	8.5 ± 2.2 *
Isobutyric acid	n.d.	3.2 ± 0.9	1.8 ± 1.2
Butanoic acid	n.d.	3.6 ± 1.2	4.9 ± 1.1
Isovaleric acid	n.d.	1.5 ± 1.30	2.7 ± 0.5

Data are expressed as mean ± SEM. Asterisk (*) indicates statistically significant differences (*p*-value < 0.05) evaluated by One-way ANOVA followed by Tukey’s test. n.d., not detected.

## Data Availability

The data presented in this study are available on request from the corresponding author.

## Supplementary Materials

The following supporting information can be downloaded at: https://www.mdpi.com/article/10.3390/nu16050588/s1, Table S1. Concentration of volatile organic compounds (VOCs), expressed as ppm, of fecal medium and Thymbra digest (FMTD), fecal slurry (FS) and fecal slurry and Thymbra digest (FSTD) samples. Data are expressed as average ± standard deviation.
