# Supplementary material for: Nutritional and Physiological Properties of Thymbra spicata: In Vitro Study Using Fecal Fermentation and Intestinal Integrity Models
Source: Nutrients. 2024 Feb 21;16(5):588. doi: 10.3390/nu16050588 (PMC10934825; doi:10.3390/nu16050588)
Supplement: Supplementary file 1 [file nutrients-16-00588-s001.zip › nutrients-2814511-supplementary.pdf]

Supplementary Table S1. Concentration of Volatile Organic Compounds (VOCs), expressed as ppm, of Fecal Medium and Thymbra Digest (FMTD), Fecal Slurry (FS) and Fecal Slurry and Thymbra Digest (FSTD) samples. Data are expressed as average ± standard deviation.

Classes	Compounds	FMTD	FS	FSTD
		Average ± St. Dev	Average ± St. Dev	Average ± St. Dev
**Alcohols** (8)	Isopropyl Alcohol	2.75 ± 0.84	0.08 ± 0.07	3.95 ± 6.83
	1-Octanol	0.07 ± 0.03	0.03 ± 0.03	0.13 ± 0.12
	1-Octanol, 2-butyl-	1.15 ± 0.13	0.11 ± 0.12	0.08 ± 0.13
	2-Decanol	0.18 ± 0.11	0.07 ± 0.02	0.16 ± 0.20
	1-Hexanol	n.d.	0.06 ± 0.01	1.27 ± 1.95
	Menthol	n.d.	11.92 ± 2.42	6.81 ± 0.80
	1-Undecanol	n.d.	0.11 ± 0.10	0.46 ± 0.67
	1-Hexadecanol	n.d.	0.78 ± 0.45	0.87 ± 0.15
**Carboxylic Acids** (9)	Acetic acid	0.26 ± 0.44	0.43 ± 0.37	0.25 ± 0.15
	Dibutyl phthalate	0.03 ± 0.03	0.02 ± 0.02	n.d.
	Butanoic acid	n.d.	1.04 ± 1.45	2.14 ± 2.20
	Pentanoic acid	n.d.	1.23 ± 1.86	6.53 ± 2.98
	Propanoic acid	n.d.	0.25 ± 0.41	0.75 ± 0.50
	Propanoic acid, 2-methyl-	n.d.	0.35 ± 0.61	0.85 ±0.66
	Butanoic acid, 3-methyl-	n.d.	1.59 ± 2.75	4.13 ± 2.46
	Heptanoic acid	n.d.	0.09 ± 0.16	2.80 ± 1.19
	Octanoic Acid	n.d.	0.16 ± 0.27	0.85 ± 0.59
**Hydrocarbons** (23)	Decane	0.99 ± 0.53	0.01 ± 0.02	n.d.
	Decane, 4-methyl-	0.08 ± 0.05	0.02 ± 0.02	0.20 ± 0.17
	Undecane, 5,7-dimethyl-	0.84 ± 0.41	0.04 ± 0.07	0.04 ± 0.06
	Undecane, 4,7-dimethyl-	1.11 ± 0.50	0.11 ± 0.12	0.19 ± 0.09
	Dodecane	1.28 ± 0.48	0.08 ± 0.15	n.d.
	Benzene, tert-butyl-	0.26 ± 0.28	n.d.	0.11 ± 0.12

	Undecane, 3,8-dimethyl-	1.44 ± 0.43	0.07 ± 0.06	0.52 ± 0.49
	Benzene, 1,2,3,5-tetramethyl-	0.29 ± 0.31	1.57 ± 1.12	0.06 ± 0.06
	Benzene, 1,2,4,5-tetramethyl-	7.66 ± 1.03	4.64 ± 1.39	4.41 ± 1.37
	Tetradecane	4.11 ± 2.14	0.33 ± 0.34	n.d.
	Hexane, 3,3-dimethyl-	0.54 ± 0.34	0.15 ± 0.13	0.18 ± 0.13
	Benzene, pentamethyl-	1.79 ± 0.67	0.71 ± 0.18	0.78 ± 0.46
	Hexadecane	2.2 ± 1.51	0.10 ± 0.17	n.d.
	Naphthalene	21.25 ± 7.10	13.19 ± 2.88	16.27 ± 8.32
	Naphthalene,1-methyl-	4.71 ± 4.58	1.08 ± 1.87	4.70 ± 2.70
	Naphthalene, 2-methyl-	7.31 ± 0.71	3.69 ± 0.63	3.40 ± 2.98
	Naphthalene, 1,2-dimethyl-	0.10 ± 0.06	n.d.	0.02 ± 0.03
	1,3-Benzodioxole, 5-propyl-	1.63 ± 1.42	0.04 ± 0.03	0.06 ± 0.06
	Toluene	0.02 ± 0.04	n.d.	0.21 ± 0.23
	Octane, 2,6,6-trimethyl-	0.23 ± 0.35	n.d.	0.50 ± 0.86
	Benzene, propyl-	0.33 ± 0.30	0.62 ± 0.04	n.d.
	Benzene, 1,3-bis(1,1-dimethylethyl)-	n.d.	0.06 ± 0.02	0.08 ± 0.12
	1-Tridecene	n.d.	1.27 ± 1.13	1.70 ± 0.29
**Indoles** (3)	Indole	3.91 ± 1.41	132.24 ± 30.49	58.58 ± 20.84
	1H-Indole, 3-methyl-	1.63 ± 1.84	6.68 ± 1.78	7.45 ± 3.09
	1H-Indole, 2,3-dihydro-4-methyl-	n.d.	0.04 ± 0.03	0.01 ± 0.01
**Others** (28)	Trimethylamine	0.40 ± 0.12	0.50 ± 0.08	0.07 ± 0.06
	Propanal, 2-methyl-	0.46 ± 0.13	0.01 ± 0.01	n.d.
	Acetone	1.34 ± 0.51	1.04 ± 0.24	1.24 ± 0.24
	Hexane, 2,3,4-trimethyl-	0.16 ± 0.11	0.12 ± 0.11	0.04 ± 0.07
	Butanal, 3-methyl-	3.55 ± 1.39	0.19 ± 0.04	n.d.
	Methyl Isobutyl Ketone	3.02 ± 1.05	3.57 ± 0.26	3.57 ± 0.45
	Decane, 2,4-dimethyl-	0.90 ± 0.42	0.59 ± 0.15	0.12 ± 0.12
	2-n-Propyl-1-heptanol	0.51 ± 0.58	0.08 ± 0.08	0.09 ± 0.16
	Benzene_1_ethyl_2_4_dimethyl	1.25 ± 0.67	0.20 ± 0.11	0.45 ± 0.07
	1-Undecene, 7-methyl-	1.02 ± 0.43	n.d.	0.04 ± 0.07
	Nonanal	1.11 ± 0.71	0.56 ± 0.27	n.d.

	1H-Indene, 2,3-dihydro-5-methyl-	1.54 ± 0.52	0.58 ± 0.06	1.11 ± 0.72
	Benzaldehyde	12.89 ± 4.55	2.12 ± 0.42	0.29 ± 0.24
	1H-Indene, 2,3-dihydro-4,7-dimethyl-	0.26 ± 0.10	0.07 ± 0.03	0.07 ± 0.02
	2-Cyclohexen-1-one, 2-methyl-5-(1-methylethenyl)-, (S)-	2.16 ± 0.82	n.d.	n.d.
	Benzaldehyde, 4-propyl-	8.64 ± 1.25	4.45 ± 0.58	5.74 ± 2.13
	Benzophenone	0.81 ± 0.13	0.04 ± 0.01	0.12 ± 0.05
	Ethanone, 1-(4-hydroxy-3methoxyphenyl)-	0.22 ± 0.11	n.d.	0.06 ± 0.02
	Dimethylamine	0.01 ± 0.01	0.01 ± 0.01	n.d.
	5-Hepten-2-one, 6-methyl-	n.d.	0.48 ± 0.18	0.47 ± 0.22
	Cyclohexene, 4-ethenyl-4-methyl-3-(1-methylethenyl)-1-(1-methylethyl)-, (3R-trans)-	n.d.	0.86 ± 0.20	1.00 ± 0.86
	1H-Indene, 2,3-dihydro-4,6-dimethyl-	n.d.	0.13 ± 0.19	0.07 ± 0.09
	3-Hexadecene, (Z)-	n.d.	0.05 ± 0.05	0.09 ± 0.11
	Dodecanal	n.d.	0.07 ± 0.13	0.74 ± 0.36
	Pentadecanal-	n.d.	0.35 ± 0.29	1.01 ± 1.06
	2-Pentadecanone	n.d.	0.19 ± 0.05	0.62 ± 0.27
	.gamma.-Dodecalactone	n.d.	0.17 ± 0.04	0.34 ± 0.14
	Tetradecanal	n.d.	0.13 ± 0.22	1.46 ± 0.29
**Phenols** (5)	Butylated Hydroxytoluene	0.72 ± 0.21	0.77 ± 0.32	1.46 ± 0.90
	p-Cresol	1.60 ± 1.37	52.90 ± 8.65	82.02 ± 13.05
	2,4-Di-tert-butylphenol	16.83 ± 5.35	2.32 ± 0.88	3.99 ± 1.47
	Phenol, p-tert-butyl	12.47 ± 18.09	0.02 ± 0.04	n.d.
	Phenol	n.d.	1.30 ± 0.29	0.21 ± 0.08
**Terpenes** (18)	.alpha.-Pinene	0.81 ± 0.29	0.10 ± 0.06	0.23 ± 0.22
	Bicyclo[3.1.1]heptane, 6,6-dimethyl-2-methylene-, (1S)-	1.01 ± 0.08	0.01 ± 0.01	0.42 ± 0.64
	1,3-Cyclohexadiene, 1-methyl-4-(1-methylethyl)-	1.29 ± 1.05	n.d.	0.01 ± 0.03
	Limonene	0.11 ± 0.13	0.06 ± 0.04	0.01 ± 0.01
	Bicyclo[3.1.0]hexane, 4-methylene-1-(1-methylethyl)-	0.14 ± 0.17	0.06 ± 0.06	n.d.
	.gamma.-Terpinene	13.99 ± 2.69	0.05 ± 0.07	0.06 ± 0.10
	Benzene, 1-methyl-3-(1-methylethyl)-	8.79 ± 4.25	0.45 ± 0.19	0.45 ± 0.24
	Linalool	1.22 ± 0.75	0.02 ± 0.03	n.d.

Caryophyllene	89.84 ± 27.09	11.47 ± 1.51	15.62 ± 8.18
3-Cyclohexen-1-methanol, 4-methyl-1-(1-methylethyl)-	8.81 ± 13.12	n.d.	1.30 ± 1.23
Humulene	2.05 ± 0.83	0.37 ± 0.03	0.39 ± 0.28
3-Cyclohexene-1-methanol, .alpha.,.alpha.,4-trimethyl-	1.52 ± 1.05	n.d.	0.63 ± 0.01
Naphthalene, 1,2,3,5,6,8a-hexahydro-4,7-dimethyl-1-(1-methylethyl)-, (1S-cis)-	3.93 ± 0.96	0.17 ± 0.03	0.14 ± 0.07
Benzenemethanol, .alpha.,.alpha., 4-trimethyl-	1.41 ± 0.48	n.d.	0.09 ± 0.04
Phenol, 2-methyl-5-(1-methylethyl)-	127.61 ± 31.18	7.53 ± 0.80	98.59 ± 32.08
Benzene, 1-methyl-4-(1-methylethyl)-	0.53 ± 0.55	0.02 ± 0.04	0.76 ± 0.62
Caparratriene	n.d.	0.30 ± 0.34	1.52 ± 1.13
Hexadecanal	n.d.	0.82 ± 0.47	2.06 ± 0.26

[1]n.d., not detected.
